# Bacterial Biofilm Inhibition in the Development of Effective Anti-Virulence Strategy

**DOI:** 10.2174/1874104501812010084

**Published:** 2018-08-31

**Authors:** Barbara Parrino, Patrizia Diana, Girolamo Cirrincione, Stella Cascioferro

**Affiliations:** Dipartimento di Scienze e Tecnologie Biologiche, Chimiche e Farmaceutiche, Sezione di Chimica e Tecnologie Farmaceutiche, Università degli Studi di Palermo, Via Archirafi 32, 90123, Palermo, Italy

There is an urgent need for new therapeutic strategies to counteract the global threat of antibiotic resistance, which has become, in recent years, one of the major public health concern [1].

An important contribution to the microbial survival in hostile environments has been given by the capability of pathogens to form sessile communities able to adhere to biotic or abiotic surfaces, known as biofilms [2].

In biofilms microbial cells are embedded in a self-synthesized matrix consisting of extracellular polymeric substance (EPS) formed by polysaccharides, proteins, lipids and extracellular DNA (e-DNA) as well as molecules originating from the host, such as mucus and DNA [3].

It was observed that bacterial cells inside the biofilm are thousand times more resistant to the conventional antibiotics than the free-living forms [4]. The biofilm antibiotic resistance is correlatable to various mechanisms involved in both cellular and community level, including, at cellular level, enzymatic resistance, chemical modification of the antibiotic target and changes in cell permeability whereas, at biofilm level, the limited penetration of the antibiotic and the presence in the deepest layers of dormant cells intrinsically resistant to conventional antibiotic treatments [5, 6].

Biofilm formation is currently one of the most relevant key virulence factor for Gram-positive and Gram-negative pathogens responsible for serious chronic infections such as chronic wound infections, pneumonia in cystic fibrosis patients, osteomyelitis, and otitis [2, 7].

Despite many efforts have been made with the aim to obtain compounds able to modulate bacterial biofilm life cycle, inhibiting biofilm formation [8-11] or dispersing pre-formed biofilms [12], biofilm formation remains the leading cause of antibiotic treatment failure, and biofilm-related infections are extremely challenging to treat [13]. The main shortcomings of scientific research in this field are the lack of *in vitro* and *in vivo* studies for clarifying the mechanisms of action and for assessing the clinical potential of the new anti-biofilm compounds.

Contrary to conventional antibiotics, most of the anti-biofilm compounds act as anti-virulence agents as they affect a virulence factor (biofilm) not interfering with the bacterial growth, hence demanding a low selection pressure for the development of antibiotic-resistance mutants.

To date the mechanisms of action of the known anti-biofilm compounds may involve:(i) the inhibition of the bacterial adhesion, which is considered the initial steps in bacterial pathogenesis [6]; (ii) the modulation of the quorum sensing (QS) system [14]; (iii) the interference with the nucleotide second messenger signaling systems [15], and (iv) the disruption of the structure of mature biofilm [12].

Sortase A (SrtA) represents an ideal target for the development of new anti-biofilm agents able to interfere with the bacterial adhesion of important Gram-positive pathogens [16].

SrtA is a transpeptidase responsible for the anchorage of surface proteins, known as microbial surface components recognizing adhesive matrix molecules (MSCRAMMs), to the cell wall envelope of Gram-positive bacteria. Many MSCRAMMs play pitoval roles in the severity of chronic *S. aureus* infections, representative examples are the protein A SpA, the fibronectin binding proteins FnbpA and FnbpB, the clumping factors ClfA and ClfB, the collagen-binding protein Cna and the serine-aspartate repeat proteins SdrC, SdrD, and SdrE [17]. Some of these proteins proved to be crucial for the biofilm formation process [18].

This transpeptidase is suitable as target for new anti-virulence agents because it is involved in the bacterial adhesion but is not essential for microbial growth. Additionally, being a membrane enzyme, it is more easily approachable by inhibitors and, considering that eukaryotic cells do not possess enzymes of sortase family, it should be easier to obtain selective inhibitor endowed with a low toxicity [19].

The role of SrtA in the biofilm formation is widely described, the overexpression of SrtA is strictly correlated with the ability of some staphylococcal strains to form biofilm, whereas loss of SrtA in five *S. aureus* clinical isolates significantly reduced this capability [19].

Other suitable targets to develop an effective anti-virulence strategy are the second messengers cyclic dimeric guanosine monophosphate (c-di-GMP) and the cyclic dimeric adenosine monophosphate (c-di-AMP) which are essential for modulating biofilm formation in many Gram-negative and Gram-positive pathogens [15].

It was reported that small organic molecules targeting c-di-GMP and c-di-AMP -related pathways are able to interfere with biofilm formation and to destroy preformed biofilm through the inhibition of the synthesis of matrix components [20].

Anti-biofilm agents can have different therapeutic application depending on their effects on the biofilm: compounds which interfere with biofilm formation can be exploited in the prophylaxis of implant surgery or for the coatings in medical devices, whereas agents able to disperse biofilm structure could be administered in combination with conventional antibiotics for the treatment of biofilm-associated infections.

Despite the growing number of new potent anti-biofilm compounds described in the past decade, the treatment of serious biofilm-associated infections still remains a significant problem. Unfortunately, no anti-biofilm compound has reached the clinical use and the most promising candidates are still in early stages of drug development, this is mainly due to the lack of *in vivo* studies [21].
